# Supplementary material for: Composition of early life leukocyte populations in preterm infants with and without late-onset sepsis
Source: PLoS One. 2022 Mar 2;17(3):e0264768. doi: 10.1371/journal.pone.0264768 (PMC8890632; doi:10.1371/journal.pone.0264768)
Supplement: S1 File — (PDF) [file pone.0264768.s001.pdf]

Composition of early life leukocyte populations in preterm infants with and without late-onset sepsis

Supporting Information

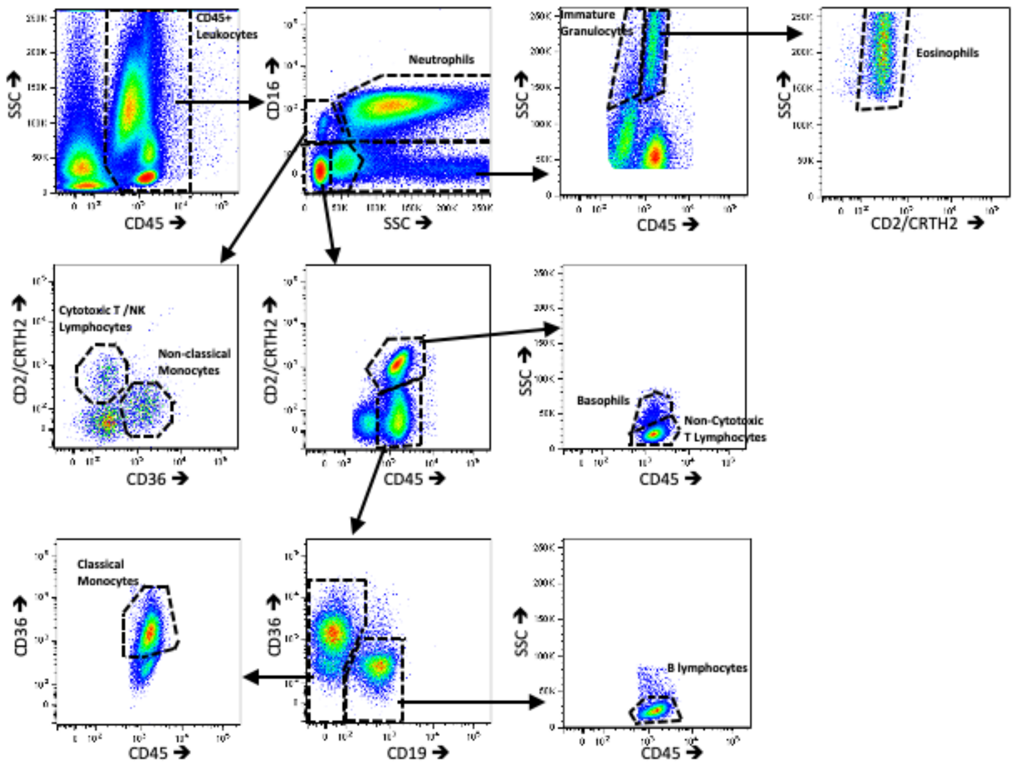

Figure S1. Leukocyte population gating strategy. Example plots of CD45^{+} leukocytes, neutrophils, basophils, immature granulocytes, eosinophils, classical and non-classical monocytes, non-cytotoxic and cytotoxic T/NK lymphocytes, and B lymphocytes from preterm infant peripheral blood.

Supplementary Table 1. Descriptive summaries of absolute counts and percentage of total CD45+ leukocyte populations in preterm infants with and without LOS. Median (95% CI) absolute counts (x 10^9/L) are presented in the first row and frequencies (%) in the second row of each timepoint.

	Any LOS n=43	Confirmed LOS n=28	Clinical LOS n=15	No LOS n=76
**CD45+ Leukocytes**				
Day 1	5.41 (4.03, 7.95)	5.30 (3.65, 7.46)	7.32 (3.84, 11.8)	5.83 (4.81, 6.68)
Day 7	6.79 (4.51, 9.08)	5.30 (4.11, 7.49)	9.08 (6.95, 11.2)	6.11 (5.19, 6.89)
Day 14	6.00 (5.05, 7.24)	5.46 (4.68, 8.24)	6.41 (5.57, 7.54)	6.29 (5.59, 7.32)
Day 21	5.73 (4.86, 6.68)	5.68 (4.30, 6.81)	6.14 (4.84, 7.86)	5.45 (4.97, 6.27)
Day 28	5.89 (4.35, 6.95)	4.70 (3.65, 6.35)	7.54 (3.68, 8.51)	5.76 (5.54, 6.49)
**Neutrophils**				
Day 1	2.15 (1.19, 3.38)	2.17 (1.17, 4.14)	2.11 (0.90, 5.05)	2.17 (1.72, 2.78)
	40.4 (36.1, 47.4)	41.2 (34.7, 48.0)	39.8 (17.9, 53.0)	38.4 (35.4, 43.3)
Day 7	1.96 (1.32, 4.38)	1.51 (0.83, 3.49)	4.38 (2.58, 6.68)	2.08 (1.55, 2.41)
	35.0 (28.6, 46.6)	28.9 (23.6, 39.4)	48.1 (31.4, 65.3)	32.8 (27.1, 35.8)
Day 14	2.48 (1.96, 3.70)	2.21 (1.31, 3.78)	2.89 (2.12, 3.73)	2.42 (2.03, 2.92)
	39.3 (35.4, 50.5)	37.5 (31.1, 50.5)	39.4 (38.1, 52.3)	38.4 (34.2, 42.6)
Day 21	2.09 (1.63, 2.46)	2.12 (1.49, 2.92)	2.02 (1.60, 2.68)	1.49 (1.25, 1.79)
	36.4 (32.7, 40.2)	37.5 (31.4, 43.9)	34.2 (31.3, 40.5)	29.5 (27.4, 32.0)
Day 28	1.77 (1.37, 2.69)	1.57 (1.07, 2.22)	3.30 (0.66, 5.38)	1.29 (1.12, 1.66)
	36.5 (28.7, 41.0)	33.3 (26.31, 40.1)	43.6 (23.0, 63.2)	23.2 (20.6, 27.1)
**Basophils**				
Day 1	0.03 (0.02, 0.04)	0.03 (0.02, 0.04)	0.02 (0.02, 0.04)	0.02 (0.02, 0.03)
	0.40 (0.30, 0.52)	0.38 (0.33, 0.59)	0.38 (0.20, 0.52)	0.40 (0.41, 0.52)
Day 7	0.03 (0.03, 0.05)	0.03 (0.03, 0.06)	0.04 (0.02, 0.07)	0.04 (0.04, 0.05)
	0.55 (0.43, 0.78)	0.64 (0.43, 0.91)	0.49 (0.30, 0.79)	0.70 (0.62, 0.83)
Day 14	0.03 (0.02, 0.04)	0.03 (0.02, 0.05)	0.03 (0.02, 0.04)	0.05 (0.04, 0.06)
	0.60 (0.39, 0.73)	0.59 (0.44, 0.80)	0.39 (0.24, 0.90)	0.70 (0.60, 0.87)
Day 21	0.03 (0.02, 0.04)	0.03 (0.02, 0.04)	0.03 (0.02, 0.05)	0.03 (0.03, 0.04)
	0.50 (0.39, 0.63)	0.48 (0.37, 0.71)	0.41 (0.25, 0.70)	0.65 (0.56, 0.70)
Day 28	0.02 (0.02, 0.04)	0.02 (0.02, 0.04)	0.03 (0.02, 0.04)	0.03 (0.03, 0.03)
	0.40 (0.36, 0.59)	0.48 (0.31, 0.73)	0.42 (0.23, 0.94)	0.60 (0.54, 0.66)
**Immature granulocytes**				
Day 1	0.10 (0.06, 0.27)	0.09 (0.05, 0.20)	0.21 (0.06, 1.59)	0.13 (0.09, 0.20)
	2.40 (1.37, 2.99)	1.81 (1.21, 2.93)	2.69 (1.97, 14.0)	2.30 (1.66, 3.15)
Day 7	0.19 (0.11, 0.30)	0.17 (0.09, 0.30)	0.22 (0.12, 0.61)	0.14 (0.10, 0.21)
	3.05 (1.91, 4.86)	2.95 (1.75, 5.75)	4.67 (1.77, 6.58)	2.20 (1.84, 2.83)
Day 14	0.18 (0.06, 0.15)	0.17 (0.09, 0.27)	0.22 (0.05, 0.28)	0.08 (0.06, 0.11)
	3.00 (1.71, 4.30)	2.68 (1.71, 5.25)	3.10 (0.80, 4.30)	1.60 (1.02, 1.96)
Day 21	0.11 (0.03, 0.13)	0.10 (0.04, 0.21)	0.11 (0.06, 0.27)	0.05 (0.04, 0.07)
	1.77 (1.09, 2.64)	1.64 (0.65, 3.52)	2.09 (0.81, 3.27)	1.10 (0.74, 1.35)
Day 28	0.06 (0.09, 0.20)	0.04 (0.03, 0.13)	0.09 (0.03, 0.30)	0.05 (0.04, 0.06)
	1.10 (0.77, 1.84)	0.90 (0.55, 1.51)	1.84 (1.05, 3.22)	0.90 (0.62, 1.28)
**Eosinophils**				
Day 1	0.02 (0.01, 0.03)	0.01 (0.00, 0.02)	0.03 (0.02, 0.07)	0.03 (0.02, 0.03)
	0.20 (0.13, 0.62)	0.14 (0.90, 0.41)	0.62 (0.19, 1.03)	0.50 (0.31, 0.69)
Day 7	0.11 (0.07, 0.15)	0.10 (0.06, 0.15)	0.15 (0.06, 0.24)	0.12 (0.09, 0.17)
	1.85 (1.00, 2.61)	1.80 (0.94, 2.61)	2.48 (0.74, 4.79)	2.40 (1.65, 2.98)
Day 14	0.16 (0.05, 0.23)	0.14 (0.04, 0.21)	0.18 (0.05, 0.40)	0.10 (0.07, 0.12)
	2.30 (1.08, 3.54)	2.52 (0.55, 3.54)	2.35 (1.76, 6.33)	1.50 (1.24, 1.96)
Day 21	0.20 (0.15, 0.32)	0.20 (0.12, 0.31)	0.20 (0.13, 0.58)	0.17 (0.13, 0.21)
	3.20 (2.47, 3.65)	3.17 (2.27, 5.80)	3.26 (2.34, 8.07)	3.00 (2.42, 4.10)

Day 28	0.16 (0.13, 0.24)	0.16 (0.11, 0.31)	0.18 (0.06, 0.33)	0.16 (0.14, 0.23)
	3.08 (2.35, 3.96)	3.09 (2.33, 4.94)	2.80 (1.94, 7.29)	3.10 (2.30, 4.09)
**Classical monocytes**				
Day 1	0.40 (0.22, 0.61)	0.28 (0.22, 0.61)	0.43 (0.14, 1.13)	0.45 (0.38, 0.53)
	6.61 (5.54, 7.56)	6.28 (4.64, 7.51)	6.94 (3.12, 9.83)	7.45 (6.22, 8.60)
Day 7	0.61 (0.48, 0.78)	0.61 (0.36, 0.84)	0.61 (0.38, 0.78)	0.59 (0.49, 0.68)
	8.75 (6.36, 11.3)	8.95 (5.85, 11.7)	8.52 (3.77, 14.0)	8.30 (6.57, 10.9)
Day 14	0.42 (0.34, 0.61)	0.39 (0.27, 0.54)	0.60 (0.32, 0.69)	0.61 (0.49, 0.71)
	7.60 (5.68, 8.38)	6.80 (4.98, 8.33)	8.14 (5.86, 10.2)	8.55 (7.35, 9.48)
Day 21	0.44 (0.32, 0.58)	0.42 (0.23, 0.58)	0.45 (0.32, 0.69)	0.39 (0.32, 0.50)
	6.90 (6.01, 8.20)	6.60 (5.61, 7.61)	8.42 (5.54, 11.3)	7.40 (6.40, 8.31)
Day 28	0.45 (0.35, 0.53)	0.41 (0.26, 0.54)	0.47 (0.35, 0.79)	0.46 (0.38, 0.54)
	7.95 (6.91, 8.55)	7.28 (6.31, 11.1)	8.07 (5.37, 9.15)	7.80 (6.74, 9.02)
**Non-classical monocytes**				
Day 1	0.05 (0.04, 0.09)	0.05 (0.03, 0.09)	0.05 (0.03, 0.15)	0.07 (0.05, 0.09)
	0.90 (0.69, 1.17)	0.79 (0.60, 1.12)	1.08 (0.67, 1.31)	0.95 (0.84, 1.27)
Day 7	0.06 (0.05, 0.10)	0.06 (0.03, 0.10)	0.06 (0.05, 0.12)	0.06 (0.05, 0.08)
	0.90 (0.60, 1.33)	0.93 (0.36, 1.62)	0.90 (0.60, 1.82)	1.00 (0.79, 1.20)
Day 14	0.08 (0.05, 0.12)	0.06 (0.04, 0.10)	0.12 (0.06, 0.15)	0.09 (0.07, 0.12)
	1.24 (0.96, 1.72)	1.05 (0.77, 1.38)	1.72 (0.96, 3.01)	1.40 (1.19, 1.60)
Day 21	0.08 (0.06, 0.13)	0.08 (0.05, 0.13)	0.12 (0.06, 0.18)	0.06 (0.05, 0.09)
	1.40 (1.14, 1.84)	1.37 (1.05, 1.84)	1.68 (1.00, 2.79)	1.10 (0.94, 1.42)
Day 28	0.08 (0.06, 0.11)	0.07 (0.06, 0.11)	0.11 (0.04, 0.20)	0.07 (0.06, 0.10)
	1.64 (1.31, 1.98)	1.68 (1.13, 2.10)	1.54 (1.10, 2.29)	1.50 (1.21, 1.70)
**Non-cytotoxic T lymphocytes**				
Day 1	0.92 (0.61, 1.09)	0.89 (0.41, 1.11)	1.02 (0.53, 1.36)	0.95 (0.83, 1.26)
	13.6 (11.7, 17.9)	14.9 (11.7, 22.1)	13.1 (7.48, 21.4)	19.5 (16.9, 21.1)
Day 7	1.10 (0.87, 1.32)	1.27 (0.87, 1.86)	0.96 (0.66, 1.31)	1.51 (1.27, 2.00)
	20.3 (13.8, 24.9)	24.9 (17.1, 32.4)	12.0 (8.13, 20.9)	27.0 (24.4, 29.9)
Day 14	1.07 (0.83, 1.38)	1.21 (0.81, 1.60)	0.86 (0.66, 1.31)	1.55 (1.23, 1.76)
	15.5 (13.5, 24.7)	16.7 (13.0, 26.3)	14.5 (12.0, 21.7)	25.2 (20.2, 27.3)
Day 21	1.11 (0.91, 1.67)	1.62 (0.94, 1.79)	1.01 (0.46, 1.17)	1.71 (1.43, 1.95)
	18.6 (14.7, 25.4)	22.9 (15.1, 27.5)	15.2 (9.39, 20.8)	30.1 (26.5, 33.0)
Day 28	1.15 (0.76, 1.42)	1.27 (0.75, 1.60)	0.93 (0.44, 1.85)	1.95 (1.63, 2.15)
	21.7 (16.6, 29.1)	23.5 (16.6, 30.1)	19.2 (9.22, 31.9)	32.4 (30.3, 35.8)
**Cytotoxic T/NK lymphocytes**				
Day 1	0.03 (0.02, 0.05)	0.03 (0.02, 0.04)	0.05 (0.03, 0.10)	0.03 (0.03, 0.05)
	0.50 (0.28, 0.77)	0.53 (0.27, 0.77)	0.64 (0.25, 1.10)	0.50 (0.46, 0.77)
Day 7	0.02 (0.02, 0.03)	0.02 (0.01, 0.03)	0.02 (0.01, 0.06)	0.03 (0.03, 0.04)
	0.40 (0.31, 0.48)	0.40 (0.23, 0.55)	0.34 (0.18, 0.65)	0.50 (0.39, 0.57)
Day 14	0.04 (0.02, 0.05)	0.03 (0.02, 0.05)	0.04 (0.01, 0.07)	0.03 (0.03, 0.04)
	0.50 (0.33, 0.75)	0.44 (0.27, 0.71)	0.74 (0.20, 1.04)	0.50 (0.47, 0.59)
Day 21	0.05 (0.04, 0.06)	0.06 (0.04, 0.09)	0.04 (0.02, 0.06)	0.04 (0.03, 0.05)
	0.90 (0.69, 1.18)	0.99 (0.70, 1.38)	0.60 (0.30, 1.13)	0.75 (0.57, 0.87)
Day 28	0.05 (0.03, 0.06)	0.06 (0.04, 0.09)	0.05 (0.03, 0.07)	0.06 (0.05, 0.08)
	1.00 (0.74, 1.27)	1.12 (0.74, 1.41)	0.75 (0.39, 1.32)	1.10 (0.80, 1.22)
**B lymphocytes**				
Day 1	0.20 (0.16, 0.30)	0.19 (0.16, 0.35)	0.25 (0.11, 0.33)	0.21 (0.18, 0.26)
	3.10 (2.50, 4.95)	3.66 (2.11, 6.79)	2.73 (2.16, 5.01)	3.85 (2.97, 4.56)
Day 7	0.22 (0.13, 0.32)	0.23 (0.13, 0.38)	0.17 (0.08, 0.35)	0.25 (0.19, 0.30)
	2.90 (2.48, 3.73)	3.18 (2.64, 5.29)	2.44 (1.07, 3.68)	3.60 (3.10, 4.08)
Day 14	0.23 (0.18, 0.31)	0.26 (0.19, 0.40)	0.19 (0.13, 0.33)	0.30 (0.22, 0.35)
	4.20 (3.18, 5.52)	4.47 (3.43, 5.79)	2.77 (2.02, 6.98)	4.25 (3.04, 5.60)
Day 21	0.28 (0.18, 0.38)	0.31 (0.18, 0.50)	0.21 (0.12, 0.38)	0.25 (0.20, 0.34)
	4.60 (3.20, 5.52)	5.01 (3.27, 7.41)	3.35 (2.46, 4.73)	4.10 (3.47, 5.33)

Day 28	0.25 (0.21, 0.35)	0.31 (0.13, 0.49)	0.23 (0.13, 0.28)	0.37 (0.23, 0.45)
	4.45 (3.21, 5.96)	5.65 (3.31, 7.93)	3.21 (2.33, 5.93)	6.40 (4.62, 7.59)

Supplementary Table 2. Basic clinical characteristics of the infants with confirmed and clinical LOS

	**Confirmed LOS n=28**	**Clinical LOS n=15**	***P* value**
Gestational age (weeks)	26.6 (25.4-28.3)	25.4 (24.7-26.4)	0.069
Birthweight (grams)	835 (690-1028)	705 (600-865)	0.119
Birthweight z-score	-0.06 (-0.75-0.21)	-0.04 (-0.98-0.65)	0.855
Males	15 (53.6)	7 (46.7)	0.755
Multiple birth	7 (25.0)	2 (13.3)	0.458
Caesarean section	16 (57.1)	9 (60.0)	>0.999
ROM	8 (28.6)	9 (60.0)	0.057
Antenatal steroids	25 (89.3)	15 (100)	0.302
Apgar <7 at 5 min	8 (28.6)	4 (26.7)	>0.999
Chorioamnionitis^	9/26 (34.6)	9/14 (64.3)	0.101
Mechanical ventilation	27 (96.4)	14 (93.3)	>0.999
Duration (hours)	177 (27-773)	393 (153-1337)	0.116
Continuous positive airway pressure	28 (100)	15 (100)	>0.999
Duration (hours)	845 (642-1243)	952 (655-1164)	0.990
IVH (grade III/IV)	1 (3.6)	3 (20.0)	0.114
Chronic lung disease	8 (28.6)	7 (46.7)	0.318
ROP (stage III/IV)^	1/25 (4.0)	1/15 (6.7)	>0.999
Length of NICU stay (days)	99 (60-117)	105 (94-164)	0.182
Mortality to 28 days	0 (0)	0 (0)	>0.999
Age at sepsis onset (days)	11 (9-16)	16 (10-20)	0.130
Highest CRP (mg/L)*	57(31-91)	26(19-45)	**0.004**

Data are expressed as median (IQR) or n (%), as appropriate. ^from available reports. * Within 72 hour of blood culture. ROM, rupture of membranes >24 hours prior to delivery; IVH, intraventricular haemorrhage; ROP, retinopathy of prematurity. CRP, C-reactive protein.

Supplementary Table 3. Descriptive summaries of adjusted absolute counts and percentage of total CD45^{+} leukocyte populations in preterm infants with and without LOS. Estimated marginal mean (95% CI) absolute counts (x 10^{9}/L) are presented in the first row and frequencies (%) in the second row of each timepoint.

	Any LOS<br>n=43	Confirmed LOS<br>n=28	Clinical LOS<br>n=15	No LOS<br>n=76
**CD45^{+} Leukocytes**				
Day 1	6.03 (5.11, 7.12)	5.30 (3.65, 7.46)	7.32 (3.84, 11.8)	5.59 (4.94, 6.32)
Day 7	6.50 (5.59, 7.57)	5.30 (4.11, 7.49)	9.08 (6.95, 11.2)	5.90 (5.25, 6.62)
Day 14	6.20 (5.38, 7.15)	5.46 (4.68, 8.24)	6.41 (5.57, 7.54)	6.51 (5.84, 7.25)
Day 21	5.90 (5.15, 6.76)	5.68 (4.30, 6.81)	6.14 (4.84, 7.86)	5.54 (4.98, 6.16)
Day 28	5.43 (4.72, 6.23)	4.70 (3.65, 6.35)	7.54 (3.68, 8.51)	5.57 (5.00, 6.19)
**Neutrophils**				
Day 1	1.57 (1.30, 1.89)	2.00 (1.44, 2.78)	1.38 (0.88, 2.17)	2.26 (1.98, 2.59)
	36.6 (32.9, 40.3)	42.3 (37.0, 47.7)	33.7 (26.3, 41.1)	40.9 (38.2, 43.7)
Day 7	1.55 (1.29, 1.87)	1.51 (1.09, 2.10)	2.25 (1.43, 3.53)	1.91 (1.66, 2.19)
	34.4 (30.8, 38.1)	33.8 (28.4, 39.1)	43.3 (36.1, 50.5)	34.7 (31.9, 37.6)
Day 14	1.93 (1.59, 2.35)	2.06 (1.48, 2.87)	2.39 (1.50, 3.82)	2.22 (1.92, 2.57)
	38.8 (35.1, 42.5)	41.1 (35.9, 46.2)	42.4 (34.8, 49.9)	39.5 (36.7, 42.4)
Day 21	1.65 (1.35, 2.02)	1.82 (1.30, 2.56)	1.89 (1.19, 3.02)	1.79 (1.53, 2.10)
	34.0 (30.3, 37.6)	36.9 (31.8, 42.0)	36.4 (29.4, 43.4)	33.0 (30.1, 35.9)
Day 28	1.60 (1.28, 2.01)	1.66 (1.16, 2.37)	2.03 (1.25, 3.31)	1.46 (1.23, 1.73)
	34.4 (30.6, 38.1)	35.3 (30.0, 40.5)	40.8 (33.8, 47.9)	28.0 (25.1, 30.9)
**Basophils**				
Day 1	0.02 (0.02, 0.03)	0.02 (0.02, 0.03)	0.02 (0.02, 0.03)	0.02 (0.02, 0.03)
	0.41 (0.35, 0.49)	0.40 (0.32, 0.49)	0.36 (0.26, 0.49)	0.42 (0.37, 0.48)
Day 7	0.03 (0.03, 0.04)	0.03 (0.03, 0.04)	0.03 (0.02, 0.04)	0.04 (0.03, 0.05)
	0.56 (0.47, 0.67)	0.54 (0.43, 0.67)	0.49 (0.36, 0.67)	0.67 (0.59, 0.77)
Day 14	0.03 (0.03, 0.04)	0.03 (0.02, 0.04)	0.03 (0.02, 0.04)	0.04 (0.03, 0.05)
	0.52 (0.43, 0.62)	0.47 (0.38, 0.60)	0.49 (0.35, 0.69)	0.68 (0.59, 0.78)
Day 21	0.03 (0.02, 0.03)	0.03 (0.02, 0.03)	0.03 (0.02, 0.04)	0.03 (0.03, 0.04)
	0.49 (0.41, 0.59)	0.45 (0.36, 0.57)	0.47 (0.34, 0.64)	0.56 (0.48, 0.64)
Day 28	0.03 (0.02, 0.03)	0.03 (0.02, 0.03)	0.03 (0.02, 0.04)	0.03 (0.03, 0.03)
	0.48 (0.40, 0.58)	0.43 (0.33, 0.55)	0.47 (0.34, 0.66)	0.51 (0.44, 0.59)
**Immature granulocytes**				
Day 1	0.15 (0.12, 0.20)	0.15 (0.10, 0.20)	0.24 (0.15, 0.38)	0.16 (0.13, 0.20)
	2.62 (2.01, 3.43)	2.41 (1.73, 3.35)	3.99 (2.53, 6.27)	2.75 (2.25, 3.34)
Day 7	0.16 (0.12, 0.20)	0.17 (0.12, 0.25)	0.17 (0.11, 0.28)	0.15 (0.12, 0.18)
	2.65 (2.03, 3.47)	2.88 (2.03, 4.06)	2.88 (1.80, 4.61)	2.48 (2.02, 3.06)
Day 14	0.14 (0.10, 0.18)	0.16 (0.11, 0.23)	0.13 (0.08, 0.22)	0.09 (0.07, 0.11)
	2.32 (1.76, 3.07)	2.69 (1.88, 3.85)	2.20 (1.31, 3.71)	1.49 (1.21, 1.85)
Day 21	0.10 (0.07, 0.13)	0.11 (0.07, 0.16)	0.11 (0.06, 0.18)	0.07 (0.05, 0.08)
	1.62 (1.21, 2.16)	1.79 (1.22, 2.62)	1.76 (1.04, 2.96)	1.11 (0.88, 1.39)
Day 28	0.07 (0.05, 0.10)	0.07 (0.05, 0.11)	0.10 (0.06, 0.17)	0.06 (0.04, 0.07)
	1.22 (0.89, 1.68)	1.22 (0.81, 1.86)	1.65 (0.94, 2.89)	0.97 (0.75, 1.24)
**Eosinophils**				
Day 1	0.02 (0.01, 0.02)	0.01 (0.01, 0.02)	0.03 (0.02, 0.05)	0.02 (0.02, 0.03)
	0.26 (0.18, 0.37)	0.19 (0.12, 0.30)	0.48 (0.26, 0.90)	0.40 (0.31, 0.52)
Day 7	0.09 (0.06, 0.12)	0.08 (0.05, 0.12)	0.12 (0.06, 0.23)	0.12 (0.09, 0.16)
	1.46 (1.03, 2.08)	1.28 (0.79, 2.05)	1.98 (1.04, 3.77)	2.02 (1.55, 2.64)
Day 14	0.09 (0.06, 0.13)	0.08 (0.05, 0.13)	0.14 (0.07, 0.28)	0.09 (0.07, 0.11)
	1.54 (1.08, 2.19)	1.28 (0.78, 2.08)	2.26 (1.11, 4.59)	1.45 (1.11, 1.91)
Day 21	0.15 (0.10, 0.21)	0.13 (0.08, 0.22)	0.20 (0.10, 0.41)	0.16 (0.12, 0.22)
	2.53 (1.77, 3.63)	2.18 (1.30, 3.65)	3.33 (1.64, 6.76)	2.79 (2.10, 3.69)

Day 28	0.15 (0.10, 0.21)	0.16 (0.09, 0.27)	0.13 (0.06, 0.28)	0.16 (0.12, 0.22)
	2.47 (1.69, 3.62)	2.60 (1.49, 4.53)	2.19 (1.03, 4.66)	2.75 (2.05, 3.70)
**Classical monocytes**				
Day 1	0.33 (0.25, 0.42)	0.31 (0.24, 0.40)	0.33 (0.23, 0.48)	0.37 (0.31, 0.45)
	5.53 (4.27, 7.17)	5.13 (3.95, 6.66)	5.50 (3.84, 7.86)	6.32 (5.23, 7.64)
Day 7	0.45 (0.34, 0.59)	0.42 (0.33, 0.55)	0.46 (0.32, 0.65)	0.43 (0.35, 0.53)
	7.62 (5.83, 9.06)	7.01 (5.43, 9.06)	7.56 (5.34, 10.7)	7.36 (6.00, 9.01)
Day 14	0.40 (0.30, 0.52)	0.33 (0.26, 0.42)	0.54 (0.38, 0.77)	0.41 (0.33, 0.50)
	6.73 (5.10, 8.87)	5.43 (4.25, 6.94)	8.92 (6.22, 12.8)	6.88 (5.59, 8.48)
Day 21	0.42 (0.32, 0.56)	0.35 (0.27, 0.44)	0.56 (0.41, 0.78)	0.36 (0.29, 0.45)
	7.13 (5.38, 9.46)	5.73 (4.51, 7.27)	9.34 (6.73, 13.0)	6.08 (4.90, 7.55)
Day 28	0.47 (0.35, 0.63)	0.46 (0.36, 0.58)	0.47 (0.34, 0.65)	0.37 (0.29, 0.46)
	7.89 (5.88, 10.6)	7.53 (5.92, 9.59)	7.74 (5.60, 10.7)	6.20 (4.96, 7.74)
**Non-classical monocytes**				
Day 1	0.05 (0.04, 0.06)	0.05 (0.04, 0.07)	0.05 (0.03, 0.08)	0.06 (0.05, 0.07)
	0.77 (0.62, 0.96)	0.80 (0.60, 1.08)	0.85 (0.56, 1.28)	1.06 (0.90, 1.24)
Day 7	0.05 (0.04, 0.06)	0.05 (0.04, 0.06)	0.06 (0.04, 0.09)	0.06 (0.05, 0.07)
	0.81 (0.66, 1.00)	0.78 (0.59, 1.05)	1.02 (0.69, 1.51)	1.04 (0.88, 1.22)
Day 14	0.07 (0.05, 0.08)	0.06 (0.05, 0.08)	0.09 (0.06, 0.14)	0.08 (0.07, 0.10)
	1.10 (0.90, 1.35)	1.02 (0.77, 1.35)	1.57 (1.04, 2.35)	1.43 (1.23, 1.68)
Day 21	0.08 (0.07, 0.10)	0.08 (0.06, 0.11)	0.10 (0.07, 0.15)	0.07 (0.06, 0.09)
	1.37 (1.13, 1.67)	1.35 (1.03, 1.78)	1.67 (1.15, 2.43)	1.25 (1.07, 1.46)
Day 28	0.08 (0.07, 0.10)	0.08 (0.06, 0.11)	0.09 (0.06, 0.13)	0.09 (0.07, 0.10)
	1.36 (1.11, 1.67)	1.40 (1.06, 1.85)	1.53 (1.05, 2.23)	1.46 (1.25, 1.71)
**Non-cytotoxic T lymphocytes**				
Day 1	0.88 (0.76, 1.01)	0.71 (0.59, 0.86)	0.93 (0.71, 1.20)	0.98 (0.88, 1.08)
	18.0 (15.3, 20.5)	14.2 (11.2, 17.2)	18.7 (14.6, 22.8)	18.3 (16.5, 20.1)
Day 7	1.24 (1.08, 1.41)	1.28 (1.07, 1.53)	0.87 (0.68, 1.11)	1.36 (1.23, 1.51)
	24.4 (21.8, 26.9)	24.6 (21.7, 27.4)	17.8 (13.9, 21.7)	25.6 (23.7, 27.5)
Day 14	1.14 (1.00, 1.30)	1.01 (0.85, 1.20)	1.05 (0.82, 1.36)	1.25 (1.13, 1.38)
	21.6 (19.0, 24.3)	19.6 (16.8, 22.4)	19.1 (15.1, 23.2)	23.5 (21.5, 25.6)
Day 21	1.17 (1.03, 1.34)	1.15 (0.95, 1.37)	0.88 (0.68, 1.13)	1.47 (1.33, 1.63)
	22.3 (19.5, 25.2)	21.7 (18.6, 24.7)	16.9 (12.7, 21.1)	27.7 (25.5, 29.9)
Day 28	1.25 (1.09, 1.45)	1.16 (0.95, 1.41)	1.02 (0.78, 1.34)	1.59 (1.42, 1.78)
	23.9 (20.8, 27.1)	22.1 (18.6, 25.6)	20.3 (15.6, 25.1)	30.4 (27.9, 32.8)
**Cytotoxic T/NK lymphocytes**				
Day 1	0.03 (0.03, 0.04)	0.03 (0.02, 0.04)	0.04 (0.03, 0.07)	0.03 (0.03, 0.04)
	0.56 (0.44, 0.71)	0.43 (0.31, 0.59)	0.71 (0.46, 1.10)	0.58 (0.48, 0.69)
Day 7	0.02 (0.02, 0.03)	0.02 (0.01, 0.03)	0.02 (0.01, 0.03)	0.03 (0.02, 0.03)
	0.37 (0.29, 0.46)	0.33 (0.24, 0.46)	0.36 (0.23, 0.55)	0.47 (0.40, 0.57)
Day 14	0.03 (0.02, 0.04)	0.03 (0.02, 0.04)	0.04 (0.02, 0.06)	0.03 (0.02, 0.04)
	0.52 (0.41, 0.65)	0.43 (0.31, 0.59)	0.60 (0.38, 0.96)	0.51 (0.42, 0.60)
Day 21	0.05 (0.04, 0.07)	0.06 (0.04, 0.08)	0.04 (0.02, 0.06)	0.04 (0.03, 0.05)
	0.89 (0.70, 1.12)	0.94 (0.68, 1.29)	0.62 (0.40, 0.96)	0.66 (0.55, 0.79)
Day 28	0.06 (0.04, 0.07)	0.06 (0.04, 0.08)	0.05 (0.03, 0.07)	0.05 (0.04, 0.06)
	0.96 (0.74, 1.23)	0.95 (0.68, 1.31)	0.75 (0.48, 1.15)	0.87 (0.72, 1.06)
**B lymphocytes**				
Day 1	0.17 (0.12, 0.24)	0.17 (0.10, 0.27)	0.15 (0.08, 0.29)	0.18 (0.14, 0.24)
	2.95 (2.11, 4.14)	2.77 (1.70, 4.51)	2.43 (1.25, 4.75)	3.11 (2.42, 3.99)
Day 7	0.18 (0.13, 0.25)	0.22 (0.15, 0.33)	0.01 (0.06, 0.17)	0.18 (0.14, 0.23)
	3.08 (2.28, 4.16)	3.65 (2.44, 5.46)	1.65 (0.95, 2.86)	3.06 (2.43, 3.85)
Day 14	0.25 (0.19, 0.33)	0.23 (0.17, 0.32)	0.23 (0.15, 0.37)	0.22 (0.18, 0.27)
	4.26 (3.21, 5.66)	3.84 (2.77, 5.32)	3.88 (2.43, 6.18)	3.69 (2.97, 4.59)
Day 21	0.27 (0.21, 0.36)	0.28 (0.21, 0.38)	0.20 (0.14, 0.29)	0.21 (0.17, 0.26)
	4.64 (3.49, 6.17)	4.70 (3.55, 6.22)	3.30 (2.25, 4.85)	3.54 (2.83, 4.42)

Day 28	0.27 (0.20, 0.37)<br>4.61 (3.35, 6.33)	0.28 (0.21, 0.37)<br>4.58 (3.45, 6.08)	0.21 (0.14, 0.30)<br>3.40 (2.32, 4.97)	0.26 (0.20, 0.33)<br>4.32 (3.38, 5.54)

Supplementary Table 4. Adjusted mean (95% CI) percentage changes overtime in absolute counts and frequency percentages of total CD45^{+} leukocyte populations in preterm infants with and without LOS. Pairwise comparisons were corrected for ten multiple tests using the Bonferroni method to maintain an overall alpha error rate of 0.05.

	**Any LOS**		**Confirmed LOS**		**Clinical LOS**		**No LOS**	
	**n=43**	***P* value**	**n=28**	***P* value**	**n=15**	***P* value**	**n=76**	***P* value**
**Neutrophils**								
Day 7 vs Day 1	-1 (-22, 26)	1.000	-24 (-49, 11)	0.436	63 (-3, 175)	0.085	-16 (-30, 2)	0.113
Day 14 vs Day 1	23 (-5, 60)	0.236	3 (-31, 54)	1.000	74 (-1, 206)	0.062	-2 (-20, 20)	1.000
Day 21 vs Day 1	5 (-21, 40)	1.000	-9 (-40, 39)	1.000	38 (-23, 145)	1.000	-21 (-36, -1)	**0.034**
Day 28 vs Day 1	2 (-25, 40)	1.000	-17 (-47, 32)	1.000	48 (-21, 175)	0.791	-35 (-49, -17)	**<0.001**
Day 14 vs Day 7	24 (-3, 59)	0.127	36 (-7, 101)	0.241	6 (-38, 84)	1.000	17 (-4, 42)	0.248
Day 21 vs Day 7	6 (-18, 38)	1.000	20 (-19, 80)	1.000	-16 (-51, 46)	1.000	-6 (-23, 16)	1.000
Day 28 vs Day 7	3 (-22, 37)	1.000	10 (-29, 69)	1.000	-10 (-49, 62)	1.000	-23 (-39, -4)	**0.009**
Day 21 vs Day 14	-15 (-33, 9)	0.696	-12 (-40, 29)	1.000	-21 (-54, 37)	1.000	-19 (-34, -2)	**0.019**
Day 28 vs Day 14	-17 (-36, 8)	0.461	-20 (-46, 21)	1.000	-15 (-52, 50)	1.000	-34 (-46, -19)	**<0.001**
Day 28 vs Day 21	-3 (-24, 24)	1.000	-9 (-38, 34)	1.000	7 (-36, 81)	1.000	-19 (-33, -1)	**0.028**
**Basophils**								
Day 7 vs Day 1	36 (3, 79)	**0.018**	35 (-10, 104)	0.369	38 (-21, 139)	1.000	59 (28, 97)	**<0.001**
Day 14 vs Day 1	25 (-5, 65)	0.241	19 (-20, 79)	1.000	37 (-23, 144)	1.000	60 (29, 99)	**<0.001**
Day 21 vs Day 1	19 (-9, 57)	0.708	14 (-24, 72)	1.000	30 (-26, 127)	1.000	31 (6, 63)	**0.004**
Day 28 vs Day 1	16 (-12, 54)	1.000	8 (-29, 64)	1.000	32 (-25, 132)	1.000	19 (-4, 49)	0.223
Day 14 vs Day 7	-8 (-30, 22)	1.000	-12 (-41, 33)	1.000	-1 (-44, 77)	1.000	1 (-19, 25)	1.000
Day 21 vs Day 7	-12 (-33, 16)	1.000	-16 (-44, 27)	1.000	-6 (-46, 64)	1.000	-17 (-34, 3)	0.140
Day 28 vs Day 7	-14 (-35, 13)	1.000	-20 (-47, 21)	1.000	-4 (-45, 68)	1.000	-25 (-40, -6)	**0.003**
Day 21 vs Day 14	-4 (-28, 26)	1.000	-4 (-36, 43)	1.000	-5 (-46, 72)	1.000	-18 (-34, 2)	0.113
Day 28 vs Day 14	-7 (-30, 23)	1.000	-9 (-40, 37)	1.000	-3 (-46, 72)	1.000	-25 (-40, -7)	**0.002**
Day 28 vs Day 21	-3 (-26, 28)	1.000	-5 (-37, 43)	1.000	2 (-41, 77)	1.000	-9 (-27, 13)	1.000
**Immature granulocytes**								
Day 7 vs Day 1	1 (-36, 60)	1.000	20 (-36, 124)	1.000	-28 (-69, 69)	1.000	-10 (-37, 29)	1.000
Day 14 vs Day 1	-11 (-45, 43)	1.000	12 (-41, 111)	1.000	-45 (-78, 36)	0.650	-46 (-62, -21)	**<0.001**
Day 21 vs Day 1	-38 (-63, 2)	0.069	-26 (-61, 43)	1.000	-56 (-82, 8)	0.101	-60 (-73, -40)	**<0.001**
Day 28 vs Day 1	-53 (-73, -19)	**0.001**	-49 (-74, 1)	0.059	-59 (-84, 5)	0.078	-65 (-77, -46)	**<0.001**

Day 14 vs Day 7	-12 (-45, 39)	1.000	-6 (-50, 75)	1.000	-24 (-69, 86)	1.000	-40 (-58, -14)	**0.001**
Day 21 vs Day 7	-39 (-62, -2)	**0.034**	-38 (-67, 18)	0.368	-39 (-74, 45)	1.000	-55 (-69, -35)	**<0.001**
Day 28 vs Day 7	-54 (-72, -23)	**<0.001**	-57 (-78, -17)	**0.003**	-43 (-77, 40)	0.804	-61 (-74, -42)	**<0.001**
Day 21 vs Day 14	-30 (-56, 10)	0.264	-34 (-64, 24)	0.640	-20 (-67, 93)	1.000	-26 (-48, 7)	0.210
Day 28 vs Day 14	-47 (-67, -15)	**0.002**	-55 (-76, -13)	**0.006**	-25 (-70, 84)	1.000	-35 (-56, -6)	**0.011**
Day 28 vs Day 21	-24 (-52, 19)	0.866	-32 (-64, 29)	0.920	-6 (-60, 119)	1.000	-13 (-39, 25)	1.000
**Eosinophils**								
Day 7 vs Day 1	461 (224, 870)	**<0.001**	573 (200, 1410)	**<0.001**	313 (38, 1130)	**0.003**	401 (227, 667)	**<0.001**
Day 14 vs Day 1	488 (233, 940)	**<0.001**	572 (197, 1420)	**<0.001**	371 (47, 1410)	**0.002**	260 (132, 459)	**<0.001**
Day 21 vs Day 1	868 (440, 1640)	**<0.001**	1050 (396, 2570)	**<0.001**	595 (120, 2100)	**<0.001**	591 (338, 990)	**<0.001**
Day 28 vs Day 1	845 (408, 1660)	**<0.001**	1270 (465, 3210)	**<0.001**	357 (38, 1410)	**0.004**	583 (323, 1000)	**<0.001**
Day 14 vs Day 7	5 (-40, 83)	1.000	0 (-56, 124)	1.000	14 (-64, 258)	1.000	-28 (-53, 11)	0.330
Day 21 vs Day 7	73 (-2, 204)	0.064	71 (-25, 289)	0.679	68 (-45, 416)	1.000	38 (-12, 115)	0.430
Day 28 vs Day 7	69 (-7, 205)	0.135	103 (-14, 381)	0.207	11 (-65, 251)	1.000	36 (-14, 116)	0.606
Day 21 vs Day 14	65 (-5, 186)	0.110	71 (-23, 281)	0.596	48 (-53, 362)	1.000	92 (24, 196)	**<0.001**
Day 28 vs Day 14	61 (-9, 185)	0.197	104 (-11, 367)	0.162	-3 (-70, 210)	1.000	89 (21, 196)	**0.001**
Day 28 vs Day 21	-2 (-44, 69)	1.000	19 (-47, 168)	1.000	-34 (-78, 96)	1.000	-1 (-36, 52)	1.000
**Classical monocytes**								
Day 7 vs Day 1	38 (-1, 92)	0.064	37 (-15, 120)	0.670	38 (-28, 163)	1.000	16 (-10, 51)	1.000
Day 14 vs Day 1	22 (-13, 70)	1.000	6 (-34, 70)	1.000	62 (-18, 220)	0.446	9 (-16, 42)	1.000
Day 21 vs Day 1	29 (-8, 81)	0.343	12 (-31, 81)	1.000	70 (-12, 229)	0.238	-4 (-26, 26)	1.000
Day 28 vs Day 1	43 (1, 102)	**0.042**	47 (-10, 140)	0.289	41 (-28, 175)	1.000	-2 (-25, 29)	1.000
Day 14 vs Day 7	-12 (-37, 23)	1.000	-23 (-52, 25)	1.000	18 (-40, 132)	1.000	-6 (-28, 22)	1.000
Day 21 vs Day 7	-6 (-33, 31)	1.000	-18 (-50, 32)	1.000	24 (-36, 138)	1.000	-17 (-37, 8)	0.455
Day 28 vs Day 7	4 (-27, 46)	1.000	7 (-34, 76)	1.000	2 (-47, 98)	1.000	-16 (-36, 10)	0.733
Day 21 vs Day 14	6 (-24, 48)	1.000	5 (-34, 69)	1.000	5 (-47, 105)	1.000	-12 (-32, 15)	1.000
Day 28 vs Day 14	17 (-16, 65)	1.000	39 (-14, 125)	0.569	-13 (-56, 71)	1.000	-10 (-31, 17)	1.000
Day 28 vs Day 21	11 (-21, 54)	1.000	32 (-18, 112)	1.000	-17 (-57, 58)	1.000	2 (-22, 32)	1.000
**Non-classical monocytes**								
Day 7 vs Day 1	5 (-25, 47)	1.000	-2 (-41, 61)	1.000	20 (-39, 136)	1.000	-2 (-24, 27)	1.000
Day 14 vs Day 1	43 (1, 103)	**0.039**	27 (-23, 111)	1.000	84 (-10, 277)	0.163	36 (4, 77)	**0.014**
Day 21 vs Day 1	78 (24, 155)	**<0.001**	69 (1, 182)	**0.044**	96 (-3, 297)	0.072	18 (-11, 57)	0.953

Day 28 vs Day 1	77 (20, 160)	**<0.001**	75 (2, 198)	**0.035**	80 (-13, 272)	0.230	38 (3, 86)	**0.021**
Day 14 vs Day 7	36 (-3, 92)	0.107	30 (-21, 115)	1.000	53 (-24, 212)	0.894	38 (6, 81)	**0.006**
Day 21 vs Day 7	69 (20, 139)	**<0.001**	73 (4, 187)	**0.025**	63 (-18, 226)	0.460	21 (-8, 59)	0.565
Day 28 vs Day 7	68 (17, 143)	**0.001**	79 (6, 203)	**0.019**	50 (-26, 204)	1.000	41 (6, 88)	**0.007**
Day 21 vs Day 14	24 (-11, 74)	0.712	32 (-19, 117)	1.000	6 (-47, 115)	1.000	-13 (-33, 14)	1.000
Day 28 vs Day 14	23 (-13, 75)	0.925	37 (-18, 128)	0.825	-2 (-52, 100)	1.000	2 (-22, 34)	1.000
Day 28 vs Day 21	-1 (-29, 39)	1.000	4 (-37, 71)	1.000	-8 (-53, 80)	1.000	17 (-10, 53)	0.962
**Non-cytotoxic T lymphocytes**								
Day 7 vs Day 1	41 (11, 80)	**0.001**	79 (36, 137)	**<0.001**	-6 (-36, 36)	1.000	39 (15, 68)	**<0.001**
Day 14 vs Day 1	30 (1, 66)	**0.033**	41 (5, 90)	**0.009**	14 (-25, 72)	1.000	22 (0, 48)	**0.041**
Day 21 vs Day 1	34 (4, 72)	**0.011**	61 (17, 121)	**<0.001**	-5 (-39, 47)	1.000	41 (16, 72)	**<0.001**
Day 28 vs Day 1	43 (11, 86)	**0.001**	62 (13, 132)	**0.002**	11 (-32, 80)	1.000	63 (33, 99)	**<0.001**
Day 14 vs Day 7	-8 (-28, 18)	1.000	-21 (-40, 4)	0.172	21 (-18, 80)	1.000	-13 (-28, 6)	0.493
Day 21 vs Day 7	-5 (-26, 21)	1.000	-10 (-33, 21)	1.000	1 (-32, 51)	1.000	1 (-17, 23)	1.000
Day 28 vs Day 7	2 (-21, 31)	1.000	-10 (-35, 25)	1.000	18 (-24, 83)	1.000	17 (-4, 43)	0.284
Day 21 vs Day 14	3 (-19, 32)	1.000	14 (-14, 50)	1.000	-17 (-44, 24)	1.000	16 (-4, 41)	0.303
Day 28 vs Day 14	11 (-14, 42)	1.000	14 (-15, 54)	1.000	-3 (-36, 48)	1.000	34 (10, 63)	**<0.001**
Day 28 vs Day 21	7 (-16, 37)	1.000	10 (-24, 33)	1.000	17 (-20, 70)	1.000	15 (-5, 39)	0.391
**Cytotoxic T/NK lymphocytes**								
Day 7 vs Day 1	-34 (-56, -2)	**0.029**	-22 (-55, 35)	1.000	-50 (-76, 6)	0.093	-18 (-39, 11)	0.683
Day 14 vs Day 1	-8 (-39, 39)	1.000	-1 (-43, 71)	1.000	-15 (-61, 85)	1.000	-13 (-36, 20)	1.000
Day 21 vs Day 1	59 (3, 144)	**0.026**	118 (26, 277)	**0.001**	-13 (-59, 84)	1.000	13 (-19, 59)	1.000
Day 28 vs Day 1	71 (7, 171)	**0.012**	120 (26, 282)	**0.001**	5 (-50, 122)	1.000	51 (6, 116)	**0.011**
Day 14 vs Day 7	40 (-6, 109)	0.175	27 (-27, 122)	1.000	70 (-22, 272)	0.580	7 (-22, 46)	1.000
Day 21 vs Day 7	141 (60, 262)	**<0.001**	180 (61, 387)	**<0.001**	74 (-18, 269)	0.390	38 (0, 91)	0.051
Day 28 vs Day 7	159 (68, 301)	**<0.001**	182 (61, 395)	**<0.001**	110 (-1, 346)	0.057	85 (31, 159)	**<0.001**
Day 21 vs Day 14	72 (16, 155)	**0.001**	120 (27, 281)	**0.001**	2 (-53, 124)	1.000	30 (-5, 78)	0.199
Day 28 vs Day 14	85 (22, 180)	**<0.001**	122 (27, 287)	**0.001**	24 (-43, 170)	1.000	73 (25, 139)	**<0.001**
Day 28 vs Day 21	18 (-27, 60)	1.000	1 (-42, 76)	1.000	21 (-43, 155)	1.000	33 (-2, 82)	0.092
**B lymphocytes**								
Day 7 vs Day 1	4 (-33, 63)	1.000	32 (-20, 118)	1.000	-32 (-66, 34)	1.000	-2 (-31, 39)	1.000
Day 14 vs Day 1	44 (-12, 137)	0.389	39 (-22, 146)	1.000	59 (-29, 256)	1.000	19 (-19, 74)	1.000

Day 21 vs Day 1	57 (-11, 176)	0.246	70 (-14, 234)	0.281	36 (-46, 243)	1.000	14 (-26, 76)	1.000
Day 28 vs Day 1	56 (-19, 199)	0.559	65 (-26, 270)	0.794	40 (-53, 319)	1.000	39 (-16, 129)	0.641
Day 14 vs Day 7	38 (-12, 118)	0.447	5 (-36, 74)	1.000	135 (16, 376)	**0.007**	21 (-15, 72)	1.000
Day 21 vs Day 7	51 (-8, 147)	0.205	29 (-28, 129)	1.000	100 (-9, 338)	0.129	16 (-22, 71)	1.000
Day 28 vs Day 7	50 (-15, 165)	0.475	25 (-37, 149)	1.000	106 (-19, 421)	0.289	41 (-9, 120)	0.282
Day 21 vs Day 14	9 (-31, 71)	1.000	22 (-25, 101)	1.000	-15 (-58, 72)	1.000	-4 (-33, 37)	1.000
Day 28 vs Day 14	8 (-35, 79)	1.000	19 (-33, 113)	1.000	-12 (-61, 96)	1.000	17 (-21, 73)	1.000
Day 28 vs Day 21	-1 (-37, 56)	1.000	-3 (-41, 61)	1.000	3 (-48, 102)	1.000	22 (-14, 74)	1.000
